# Physical activity referral to cardiac rehabilitation, leisure centre or telephone-delivered consultations in post-surgical people with breast cancer: a mixed methods process evaluation

**DOI:** 10.1186/s40814-018-0297-1

**Published:** 2018-06-01

**Authors:** Gill Hubbard, Anna Campbell, Abi Fisher, Michelle Harvie, Wendy Maltinsky, Russell Mullen, Elspeth Banks, Jackie Gracey, Trish Gorely, Julie Munro, Gozde Ozakinci

**Affiliations:** 1 Department of Nursing, University of the Highlands and Islands, Centre for Health Sciences, Old Perth Road, Inverness, IV2 3JH Scotland UK; 2 School of Life Science, Sport and Social Science, Edinburgh Napier University, Edinburgh, EH11 4B Scotland UK; 3 Department of Behavioural Science and Health, University College London, 1-19 Torrington Place, London, WC1E 6BT England UK; 4 Prevent Breast Cancer Research Unit, The Nightingale Centre, Wythenshawe Hospital, Manchester, England UK; 5 Division of Health Research, Rural Health & Wellbeing, University of the Highlands and Islands, An Lòchran, Inverness Campus, Inverness, IV2 5NA Scotland UK; 6 Highland Breast Centre, NHS Highland, Raigmore Hospital, Old Perth Road, Inverness, IV2 3UJ Scotland UK; 7 National Cancer Research Institute, Clinical Studies Group, Angel Building, 407 St John Street, London, EC1V 4AD England UK; 8 School of Health Sciences, Ulster University, Jordanstown campus, Shore Road, 7, Newtown Abbey, County Antrim, BT37 OQB Northern Ireland UK; 9 School of Medicine, Medical & Biological Sciences, University of St Andrews, North Haugh, St Andrews, KY16 9TF Scotland UK

**Keywords:** Breast cancer, Health behaviour, Complex intervention, Physical activity, Cancer survivorship, Rehabilitation

## Abstract

**Background:**

Physical activity (PA) programmes effective under ‘research’ conditions may not be effective under ‘real-world’ conditions. A potential solution is to refer patients to existing PA community-based PA services.

**Methods:**

A process evaluation of referral of post-surgical patients with early-stage breast cancer to cardiac rehabilitation exercise classes, leisure centre with 3-month free leisure centre membership or telephone-delivered PA consultations for 12 weeks. Quantitative data were collected about PA programme uptake and reach, patient engagement with the PA programme, delivery and fidelity and PA dose. Qualitative data were collected about patient experiences of taking part in the PA programmes. Audio-recorded qualitative interviews of participants about the programmes were analysed thematically. Quantitative data were reported descriptively using means and SD.

**Results:**

In Phase I, 30% (*n* = 20) of eligible patients (*n* = 20) consented, 85% (*n* = 17) chose referral to leisure centre, and 15% (*n* = 3) chose cardiac rehabilitation. In Phase II, 32% (*n* = 12) consented, 25% (*n* = 3) chose leisure centre and 75% (*n* = 9) chose telephone-delivered PA consultations. Walking at light intensity for about an hour was the most common PA. All Phase I participants received an induction by a cardiac rehabilitation physiotherapist or PA specialist from the leisure centre but only 50% of Phase II participants received an induction by a PA specialist from the leisure centre. Four themes were identified from qualitative interviews about programme choice: concerns about physical appearance, travel distance, willingness to socialise and flexibility in relation to doing PA. Four themes were identified about facilitators and barriers for engaging in PA: feeling better, feeling ill, weight management, family and friends.

**Conclusions:**

The current community-based PA intervention is not yet suitable for a definitive effectiveness randomised controlled trial. Further work is needed to optimise PR programme reach, PA dose and intervention fidelity.

**Trial registration:**

ISRCTN11183372.

**Electronic supplementary material:**

The online version of this article (10.1186/s40814-018-0297-1) contains supplementary material, which is available to authorized users.

## Background

Breast cancer is the most common cancer among women worldwide, with an estimated 1.67 million new cancer cases diagnosed in 2012 (25% of all cancers) [1]. In the United Kingdom (UK), three quarters of the 53,696 people diagnosed with breast cancer each year survive for at least 10 years [2]. Consequently, research concerning the longer-term psychosocial and physical health of people treated for breast cancer is important.

An increasing body of evidence has linked post-diagnosis physical activity (PA) to length and quality of breast cancer survivorship, with results summarised in meta-analyses and systematic reviews [3–6]. Benefits of PA include improvements in cardiorespiratory fitness, body composition (including muscle mass and bone health), strength and flexibility, body image, self-esteem, mood, stress, depression, anxiety, nausea, fatigue and pain. Fear of recurrence is one of the main causes of distress in cancer survivors [7] but PA trials have not examined if being active addresses these fears. Professional bodies in different countries, including the UK, have published detailed PA prescription guidelines for people with cancer [8–12]. These bodies concur that unless advised otherwise, all cancer survivors should aim to meet national PA recommendations for the general public (currently ≥ 150 min of moderate intensity aerobic activity per week, plus two sessions of muscular strength and endurance and some daily flexibility/balance exercises). A concern for breast cancer clinical teams is that only 16% of breast cancer survivors are meeting PA recommended guidelines [13], and sedentary time remains high in the first year following treatment for breast cancer [14]. Therefore, patients need effective evidence-based programmes to support them to increase PA.

To date, the majority of PA trials in cancer have evaluated interventions under research (e.g. group exercise class led by a researcher) rather than ‘real-world’ settings [15]. Yet, PA programmes that are effective under research conditions are not translated in practice and PA programmes are not routinely part of the standards of care provided to people following a cancer diagnosis [16]. An advantage of using existing community-based PA services is that an infrastructure already exists but should only be recommended if there is evidence that they are clinically effective and uptake in the target population will be high. Determining if existing community-based PA services are likely to be used by people with cancer to aid their recovery is especially relevant in the UK where one of the leading cancer charities is rolling out a national ‘physical activity offer’ to people recovering from cancer that includes use of existing community services [17]. There are a range of existing PA services including cardiac rehabilitation exercise classes for people with coronary heart disease, and leisure centres and personal trainers for the general public. If people with breast cancer are offered a choice of these existing PA programmes, they may perceive increased control over their options and as proposed by most social cognitive models of human behaviour, offering choice will improve motivation to change behaviour [18, 19].

Cardiac rehabilitation, which is widely available throughout the UK and in other countries, includes supervised circuit classes for people recovering from coronary heart disease. A core component of cardiac rehabilitation is PA and exercise so that people recovering from cardiovascular disease increase overall daily energy expenditure to achieve good cardiovascular health [20]. A British Heart Foundation audit found that at 12 months after participation in cardiac rehabilitation there was a 14 percentage point increase in the number of people exercising five or more times a week for 30 min and a 23 percentage point reduction in those who rarely/never took exercise [21]. Current guidelines for cardiac rehabilitation recommend use of *generic* evidence-based behaviour change techniques (BCTs) to support improvement in PA such as motivational counselling (active listening skills, empathy and open questioning), goal setting and instructing/coaching [22]. Hence, cardiac rehabilitation exercise classes using BCTs could also be relevant to people with cancer to increase PA. A core component of cancer rehabilitation is PA and exercise [8–12], and a recent study suggests that referral of people with colorectal cancer to cardiac rehabilitation exercise classes is feasible and acceptable [23]. Referral to cardiac rehabilitation exercise classes of people with breast cancer, however, has not been previously investigated.

Another existing PA service is exercise referral schemes (ERS) [24]. Most ERS operate out of community leisure centres to provide health coaching, exercise consultations, motivational interviews, community-based gym and exercise classes, walking and/or gardening activities. ERS are highly heterogeneous but typically involve health professional referral of a patient to a leisure centre, agreement of a PA programme with a leisure centre instructor and discounted access to the leisure centre for 10 to 12 weeks [25]. A review of eight randomised controlled trials of ERS found an increased number of participants who achieved 90–150 min of PA of at least moderate intensity per week (pooled relative risk 1.16, 95% confidence intervals 1.03 to 1.30) compared with controls [26]. It remains unclear if similar increases in PA could be achieved in people with breast cancer referred to a similar scheme.

Travel and distance is a barrier to attending PA exercise classes for people recovering from treatment for breast cancer [27]. Cardiac rehabilitation exercise classes and leisure centres may not be easily accessible to some people with cancer because they live in remote and rural areas. However, PA specialists located in leisure centres could potentially deliver telephone-based consultations and, in doing so, offer the unique advantages of increased convenience and access [28, 29]. A recent systematic review identified seven studies of telephone-delivered PA programmes for people with breast cancer, with evidence of a small to moderate effect on quality of life [29]. However, the review identified only two studies including people with breast cancer who were on adjuvant treatment, and hence, it remains uncertain if patients on treatment for breast cancer would engage with this type of PA programme. Motivational interviewing was used in several of the studies included in the review [29]. Motivational interviewing is recognised as a useful translation of self-determination theory (SDT) [30]. According to SDT, conditions that support a person’s basic psychological needs, which is their need for ‘autonomy,’ (feeling of being the origin of behaviour), ‘competence’ (feeling of being effective) and ‘relatedness’ (feeling of being understood and cared for by others), foster the most volitional and intrinsic forms of motivation for initiation and long-term maintenance of health behaviours including PA [31]. A growing body of empirical work has shown that SDT-based interventions are effective in augmenting changes in level of PA [32], but SDT-based interventions have rarely been tested in ‘real-world’ conditions or in people with cancer.

### Aims

In this paper, the findings of a mixed methods process evaluation of a PA intervention for people after surgery for breast cancer is reported. The main aim of the study was to understand if referral of people after surgery for breast cancer to the following existing community-based PA services—cardiac rehabilitation exercise classes, the local leisure centre with 3-month free membership, or to telephone-delivered PA consultations—is feasible to implement and if these PA programmes are acceptable to people with breast cancer. Medical Research Council guidance about process evaluation of complex interventions highlights the importance of investigating how interventions are delivered by practitioners and how patients engage with interventions as a means of understanding the implementation and functioning of the intervention in practice [33]. PA interventions are often poorly described, yet explicit reporting of what actually happens in a PA intervention is essential to the interpretation, translation and implementation of research findings into clinical practice [34]. These types of evidence are particularly useful when an intervention fails to achieve intended theorised effects because it may be explained by lack of engagement or poor delivery [25]. In addition to quantifying patient engagement and practitioner delivery, understanding patients’ motivations and barriers to PA will also help in the design of more effective interventions [25, 33]. This paper focuses on the following study’s objectives: (1) quantify PA programme uptake and reach, patient engagement with the PA programme, PA programme delivery and fidelity and PA dose and (2) qualitatively explore patient experiences of taking part in the PA programmes. There is lack of consensus about reporting the effectiveness of interventions in preliminary work conducted before a full definitive trial [35]. In this paper we do not report outcomes because we did not include a control group to evaluate intervention effects, and the study was not powered to determine meaningful differences in health outcomes pre- and post-intervention.

## Methods

### Design

The study was conducted in two phases. In Phase I, the referral of patients to cardiac rehabilitation exercise classes or to the local leisure centre with 3-month free membership was investigated. Phase I findings were used to inform which PA programmes would be included in Phase II. In Phase I, travel and distance was the most common reason why eligible patients were not willing to participate in the study. In Phase II, we aimed to improve intervention reach by removing this barrier to participation. Hence, in Phase II, we included the referral of people after surgery for breast cancer to telephone-delivered PA consultations. In Phase I, the majority of participants chose referral to the leisure centre with 3-month free membership so we only included this PA programme in Phase II. We did not include a comparison group such as a usual care group to act as a control group in either Phase I or II because the main aim of the study was to gather evidence about the implementation of the three different PA programmes in practice.

### Setting

Recruitment took place over 6 months in Phase I and 3 months in Phase II at one UK hospital serving an urban and rural population. The three different PA programmes (cardiac rehabilitation exercise class, leisure centre with 3-month free membership, telephone-delivered PA consultations) were delivered by local practitioners (either health professional delivering the local cardiac rehabilitation exercise classes or PA specialists from the local leisure centre).

### Intervention

Phase I participants were referred to either a local cardiac rehabilitation exercise class or to a local leisure centre with 3-month free membership depending on participant choice. Phase II participants were referred to a local leisure centre or to telephone-delivered PA consultations that were delivered by a PA specialist from the leisure centre depending on participant choice. As explained above, the cardiac rehabilitation exercise class was not offered in Phase II because few participants chose this PA programme in Phase I. A logic model for the intervention, which was informed by the research team’s previous work, such as the use of cardiac rehabilitation in colorectal cancer patients [23], discussions with local PA service providers, the local breast cancer care team and the literature is available in Additional file 1.

#### Cardiac rehabilitation exercise class

Participants were referred to an existing cardiac rehabilitation exercise class. The cardiac physiotherapist informed the research team that the British Association of Cardiovascular Prevention and Rehabilitation (BACPR) guidelines were followed when delivering the exercise classes including goal setting and use of motivational interviewing techniques [36]. At a face-to-face induction with a cardiac rehabilitation physiotherapist, participants were given an initial fitness assessment with incremental shuttle walk test. Specific exercises and intensities that the participant was to do in the class were discussed at induction. Participants attended a 1-h cardiac rehabilitation circuit exercise class in a gym at the local hospital once a week for 12 weeks. A cardiac physiotherapist and a physiotherapy assistant delivered the class. Exercise took place in a group setting alongside patients recovering from coronary heart disease. No changes to the cardiac rehabilitation programme were made to accommodate cancer patients. Nonetheless, the physiotherapists had attended an additional 1-day educational course delivered by a cancer and exercise specialist (AC) about PA for people recovering from breast cancer for the purposes of the study. Participants could attend health education sessions which took place after the exercise class and included general health advice (e.g. diet, exercise, relaxation), alongside cardiac specific sessions (e.g. medications).

#### Leisure centre with 3-month free membership

This PA programme was designed to be similar to typical ERS [25]. Participants were given a 3-month free leisure centre membership at one of the local leisure centres in the same city as the hospital where they were treated for breast cancer, providing them with free access to a range of fitness classes, gym and swimming pool. During an initial face-to-face induction with a PA specialist at the leisure centre, the participant received a standard health check by completing the PA Readiness Questionnaire (PAR-Q) [37] followed by agreement of an individual PA programme, including goal setting. After the face-to-face induction, there were no mandatory consultations with a leisure centre PA specialist during the remaining 12 weeks of the PA programme. PA specialists were qualified to Register of Exercise Professionals (REPs) Level 4 in Cancer and Exercise (www.canrehab.co.uk) and had attended an additional 1-day educational course delivered by a cancer and exercise specialist (AC) about PA for people recovering from breast cancer for the purposes of the study. In Phase II only, participants were also given a pedometer (2D G-sensor) to monitor step count each day.

#### Telephone-delivered PA consultations

PA specialists were the same people who were delivering the leisure centre with 3-month free membership PA programme described above. Participants initially met face-to-face with a PA specialist at the leisure centre for PA induction. At induction, participants received a health check by completing the PAR-Q [37] and an individual exercise programme was planned. During the remaining 12 weeks of the PA programme, participants had a weekly telephone-delivered PA consultation with the PA specialist. All PA consultations were conducted in accordance with self-determination theoretical (SDT) techniques [38] (see Table 1 for list of techniques). The PA specialists had attended a 1-day education event delivered by a health psychologist (WM) about motivational interviewing, which is recognised as a useful translation of SDT [30, 39]. Participants were also given a pedometer (2D G-sensor) to monitor step count each day. In line with SDT, research has shown that pedometer use leads to: increased ‘autonomy’ (through supporting tailoring of PA), increased ‘competence’ to achieve number of steps (through providing feedback) and ‘relatedness’ (surveillance of participants’ steps can help participants to feel observed and supported) [40]. Some participants were given free 3-month leisure centre membership too, although this was not a planned part of the intervention.

### Participants and recruitment

Participants were screened for eligibility at a multi-disciplinary team meeting (MDT). At each MDT, it was agreed by the clinical team which patients were eligible for the study and could therefore be approached at an out-patient appointment about the study by a breast surgeon or nurse specialist. Reasons for ineligibility were recorded at the MDT. Study information was given to eligible patients approximately 2 weeks after surgery at an out-patient appointment by a breast surgeon or nurse specialist. A researcher contacted by telephone participants who indicated willingness to participate and arranged a face-to-face meeting to discuss the study, confirm willingness to participate and obtain written informed consent.

#### Inclusion criteria

Female and male patients were eligible if they were aged 16 years or over and were recovering from surgery for early-stage (stages I–IIIA) breast cancer or ductal carcinoma in situ (DCIS). Patients were eligible if they were receiving any adjuvant treatment or had finished adjuvant treatment and were living within a 35-mile radius of the PA intervention site. Travel is a known barrier to participation in interventions [41] and the local NHS provides travel claims for those living in a > 35-mile radius of the hospital. This inclusion criterion was removed in Phase II because participants could choose telephone-delivered PA consultations, which removed travel and distance as a barrier to participation in the study.

#### Exclusion criteria

Participants were ineligible if any of the above criteria were not met and if the MDT decided that the patient lacked capacity to give informed consent or that there were medical or psychological reasons that would prevent patient adherence to a PA intervention. To facilitate MDT decision-making about eligibility, the research team delivered a presentation about PA contraindications [9] to the MDT prior to recruitment. No formal medical assessment was conducted with the patient because according to international experts ‘this would create an unnecessary barrier to obtaining the well-established health benefits of exercise for the majority of survivors, for whom metastasis and cardiotoxicity are unlikely to occur’([9]:1412). Other reasons for exclusion were that the patient was scheduled to have further surgery in the next 12 weeks and the patient had no access to a landline or mobile telephone (Phase II only).

### Sample size

No formal sample size calculation to power the study was performed. In Phase I, we used routine hospital data in the site where the study was conducted about the total number of patients having surgery for early-stage breast cancer to estimate sample size. We estimated that 140 patients over a 6-month period with early-stage breast cancer would be screened for eligibility and 56 (40%) would be eligible. Using data from a previous PA study carried out in the same country (i.e. Scotland) with people with early-stage breast cancer [41], we estimated that 15 (27%) eligible patients would consent to the study. In Phase II, we used Phase I data to estimate sample size. We estimated that 70 patients with breast cancer over a 3-month period would be screened for eligibility, 42 (60%) would be eligible and 14 (33%) would consent.

### Quantitative implementation assessments

Table 2 provides an overview of data collected for quantitative PA programme implementation assessments. These assessments drew upon MDT, leisure centre or cardiac rehabilitation routine service databases or upon forms specifically designed for the purposes of the study and completed by a member of the MDT, leisure centre PA specialists, cardiac rehabilitation physiotherapists and collated by a researcher. Data were also gathered from participants by questionnaires and diaries.

**Table 2:** Implementation assessments

Quantitative implementation assessments	PA programme activity	Indicator	Data source
Uptake and reach	Patient referral to PA programme	% of patients screened for participation % of patients who were eligible % of patients consenting % of participants dropping out of the PA programme Reasons for excluding patients Reasons why eligible patients did not wish to participate Mean age of participants % of participants on/off treatment	MDT records Researcher records
Engagement and fidelity	Providing choice of PA programme, Induction Attendance SDT-based motivational interviewing, Behaviour Change Techniques (e.g. goal-setting, monitoring)	*Participant choice of PA programme:* % choosing cardiac rehabilitation exercise classes % choosing local leisure centre with 3-month free membership % choosing telephone-based PA consultations *Cardiac rehabilitation:* Number of participants receiving induction Number of exercise classes attended by participants Leisure Centre with 3-month free membership: Number of participants receiving induction Number of visits to leisure centre over study duration % of participants taking out leisure centre membership at end of the study *Telephone-delivered PA consultations:* Number of participants receiving induction Number of consultations delivered % of SDT-based techniques used Mean score psychological needs met by PA programme	Researcher records Routine cardiac rehabilitation service records PA specialist records Leisure centre records Participant questionnaire (Perceived Environmental Supportiveness Scale)
PA dose		Frequency Total number of PA sessions (defined as an occasion when the participant did any type of PA) Intensity Mean intensity of PA sessions Time Mean minutes per week spent of PA sessions Type % of PA sessions categorised by type, e.g. walking, jogging, cycling, etc. *Other:* Mean daily step count	Participant PA diaries

#### Reach and uptake

To quantify PA programme reach and uptake, the following data were gathered: (1) A researcher collected screening, eligibility, consent, and drop-out rates; (2) Reasons for an MDT excluding patients using the eligibility criteria were recorded at the MDT; (3) Reasons for non-participation of eligible patients were recorded using free text by the breast surgeon or nurse specialist at an out-patient appointment when patients were first approached about the study or by a researcher who telephoned patients if they were interested in finding out more about the study before making up their mind whether to participate; (4) Participants’ age and if they were receiving adjuvant chemotherapy or radiotherapy at the time they were referred to a PA programme were retrieved from MDT records.

#### Engagement and fidelity

Participant choice of PA programme was recorded. It was not possible to integrate standardised assessments of patient engagement or intervention fidelity for the different PA programmes. Instead, the following data were collected from each PA programme:

##### Cardiac rehabilitation exercise class

The number of participants receiving the face-to-face induction and participant attendance at each weekly cardiac rehabilitation exercises class for 12 weeks was objectively measured from routine cardiac rehabilitation service records.

##### Leisure centre with 3-month free membership

The number of participants receiving the face-to-face induction was recorded from PA specialist records. Leisure centre attendance was objectively measured from swipe-card membership entry at the leisure centre for the study duration (Phase I = 12 months; Phase II = 8 months). The proportion of participants taking out leisure centre membership at the end of the study was obtained from leisure centre records.

##### Telephone-delivered consultations

The number of participants receiving the face-to-face induction and the total number of telephone-delivered PA consultations for each participant were self-reported by the PA specialists delivering the consultations. PA specialists self-reported their use of SDT-based techniques during each telephone-delivered PA consultation (Table 1). In addition, participants completed the Perceived Environmental Supportiveness Scale, which is a 15-item valid and reliable SDT-informed instrument to measure the extent to which participants perceive that their three basic psychological needs (autonomy, competence and relatedness) are being met by a behavioural change intervention [42].

#### PA dose

Participants completed a paper diary specifically designed for the study for 12 weeks during the PA programme. A researcher provided guidance on how the diary should be completed at the meeting when the participant provided written informed consent. For each day, participants recorded the following information about frequency, intensity, time and type (FITT) parameters:

- *Frequency:* Each ‘PA session’ was recorded. A session was defined as an occasion when the participant did any type of PA. Participants were informed that a PA session could include, for instance, a brief walk, home-based exercise as well as participation in a class at the leisure centre, cardiac rehabilitation and could be of any duration.
- *Intensity:* The Borg scale (range 6–20 with 20 being the hardest) [43], which is a validated measure of intensity, was used to record intensity for each PA session.
- *Time*: Duration in minutes for each PA session was recorded.
- *Type:* Type of PA for each PA session was recorded. During analysis, type of PA was categorised by a researcher using the following eight categories: walking; jogging/running; cycling; other cardiovascular (e.g. spinning, running machine, aerobic classes), resistance or flexibility exercises (which could be conducted either at home or in a leisure centre); swimming or housework.

In Phase II, participants were also given a pedometer so that they could monitor daily pedometer (2D G-sensor) recorded steps over the 12-week PA programme.

### Qualitative study component: participant views of the PA programme

#### Sampling

In Phases I and II, all participants were invited for interview with a researcher at the end of the 12-week PA programme.

#### Procedures

All participants were contacted by telephone to arrange an interview. A face-to-face or telephone digitally recorded interview (depending on participant preference) approximately 2 weeks after the PA programme in Phases I and II was arranged. Participants could choose whether to have the face-to-face interview take place in their own home or at the university. Informed consent interview participation was obtained when they first provided written informed consent at the beginning of the study. Verbal consent was obtained from each individual before the interview and actual recording took place. All interviews were on a one-to-one basis with the researcher.

#### Schedule

A semi-structured interview schedule was used because it allows flexibility in what sequence questions are asked, and how particular issues might be followed up and developed with different interviewees. The schedule was developed to cover factors influencing participants’ choice of PA programme, factors that facilitated and impeded engagement in the PA programme and experiences of the programme. Hence, the schedule was developed to reflect the research aims but was not too prescriptive so that the researcher could probe issues that emerged during the interview.

### Analysis

Quantitative data about PA programme implementation were analysed descriptively. Descriptive statistics were calculated for reach and uptake (e.g. screening, eligibility, consent and drop-out, reasons for ineligibility and declining participation, age and on/off treatment), engagement and fidelity (e.g. PA programme choice, PA programme attendance/consultations, SDT-based techniques), PA dose (frequency, intensity, time and type) and reported as *n* (%) for categorical data and mean (Standard deviation [SD]) for continuous data. Qualitative data about the PA programmes were analysed thematically. Audio-recorded qualitative interviews were transcribed verbatim and analysed thematically using the Framework approach [44], which is a rigorous method providing a structure within which qualitative data are organised and coded and themes are identified. In brief, a researcher (GH) became familiar with the interview transcript data by reading and rereading transcripts and assigning interview data (sentences and paragraphs) to two main themes, which were (1) choice of PA programme and (2) barriers and facilitators for engaging in PA. Second, subthemes were identified for each of these two main themes and a narrative summary of coded data was made under each subtheme. Finally, the researcher referred to the original data to ensure that participant accounts were accurately presented in this paper. Quotations to illustrate themes are available in Additional file 2. The observed effect of the PA programmes is reported descriptively using mean and SD pre- and post-intervention for the following variables: PA, self-efficacy for PA, quality of life, fatigue, and fear of recurrence.

## Results

### Reach and uptake

Participant flow throughout the study is available in Additional file 3. The screening rate in Phases I and II was > 90% (Phase I *n* = 158 (100%); Phase II *n* = 68 (94%)); the eligibility rate was 42% (*n* = 67) and 54% (*n* = 37), respectively; the consent rate was approximately 30% (Phase I *n* = 20 (30%); Phase II *n* = 21 (32%)) and the drop-out rate was 5% (*n* = 1) and 8% (*n* = 1), respectively. In Phase I, 63% (*n* = 57) were ineligible because they lived > 35-mile radius of the hospital and 17% (*n* = 8) of eligible patients did not wish to participate because of distance. Hence, in Phase II, we removed travel and distance as barriers to participating in a PA programme by giving patients choice of receiving telephone-delivered PA consultations or attending the leisure centre. In Phase II, 45% of screened patients were deemed ineligible to participate in a PA programme for health reasons (e.g. poor wound healing) by the MDT. Seventeen percent and 32% of eligible patients in Phases I and II, respectively, were not interested in participating in the study.

In both phases, the average age of consenting patients was 57 years (Phase I: range 38–77 years; Phase II: (range 43–77)). In Phases I and II, 70 and 91%, respectively, of participants were receiving adjuvant therapy when they started the PA programme. In Phase I, seven of the 20 participants were receiving chemotherapy when they started the PA programme and seven were receiving radiotherapy (with two of these having completed neo-adjuvant chemotherapy). In Phase II, two of the 12 participants were receiving chemotherapy (with radiotherapy to follow) and nine were receiving radiotherapy (one having completed neo-adjuvant chemotherapy).

### Engagement and fidelity

In Phase I, 17 (85%) participants chose referral to the leisure centre with 3-month free membership, and three (15%) chose cardiac rehabilitation exercise classes. In Phase II, three (25%) participants chose the leisure centre with 3-month free membership and nine (75%) chose telephone-delivered PA consultations. In Phase I, all three participants choosing cardiac rehabilitation exercise classes received the face-to-face induction. One participant attended all 12 weekly classes, one attended 11 out of 12 classes and one did not attend any classes. In Phase I, all 17 participants choosing the leisure centre with 3-month free membership received the face-to-face induction. According to leisure centre membership records, 11 out of 17 Phase I participants had leisure centre membership. Minimum and maximum leisure centre attendance of the 11 participants with leisure centre membership between 1 July 2015 and 1 July 2016 (i.e. during the 12 month study) was 0 and 65 times. In Phase II, nine out of 12 participants, had leisure centre membership (some who chose telephone-based PA consultations had leisure centre membership). Four participants already had leisure membership prior to admission to the study. Provision of free leisure centre membership was expected to be given to those who chose the leisure centre but it was not part of the telephone-based PA consultation programme. Nonetheless, five participants, including two who chose the telephone-based PA consultation programme, were given 3-month free leisure centre membership during Phase II by the PA specialists. Minimum and maximum leisure centre attendance of the nine participants with leisure centre membership between 1 August 2016 and 30 April 2017 (i.e. 8 months) was 0 and 14 times. None of the five participants who were provided with a free 3-month leisure centre membership took out paid membership immediately after the study.

In Phase II, two out of the three participants who chose the leisure centre with 3-month free membership had the face-to-face induction (data missing for one participant) (Table 3). Four out of the nine participants who chose the telephone-based PA consultations had the face-to-face induction. The average number of participant contacts with a PA specialist (including messages left on the telephone) over the 12-week programme was six. All nine participants who chose weekly telephone-based PA consultations had ≤ 4 telephone PA consultations.

**Table 3:** Phase II face-to-face and telephone consultations or email correspondence

Participant ID	Face-to-face	Telephone	Answerphone message	Email	Total number of contacts (including messages)
Participants choosing face-to-face PA consultations^a^					
101	4	0	2	0	6
107	4	1	0	3	8
Participants choosing telephone PA consultations					
109	0	1	1	0	2
110	1	0	2	1	3
105	0	3	1	2	6
103	0	3	0	2	5
102	2	4	0	0	6
108	1	4	0	1	6
106	0	4	0	2	6
111	0	2	2	1	5
112	3	1	0	0	4
Total	15	23	8	12	57

^a^Data missing for one participant

Out of the 23 telephone-delivered PA consultations, 14 (61%) SDT-based technique self-report questionnaires were completed by a PA specialist. Table 1 shows if the SDT-based technique was reported to have been used during the telephone-delivered consultation. PA specialists reported that they used SDT-based techniques to foster ‘autonomy’ in over 78% of the telephone-based consultations delivered. Techniques to foster ‘competence’ were not used to the same extent. For example, discussing individualised goals was used in 64% of the telephone-delivered consultations. Most techniques to foster ‘relatedness’ were used in most telephone-delivered consultations (78–100%).

**Table 1:** SDT techniques used during telephone consultations

Autonomy:	Use (range 0–14)
• Offering clear reasons to become more active?	10 (71%)
• Giving information to support decisions on different types of activity?	13 (93%)
• Give them a choice, and various options for being more active?	13 (93%)
• Encouraging enjoyment of PA by choosing activities that participants like doing.	12 (86%)
• Avoid coercion and persuasion? Encourage participant to make their own choices? (e.g. *avoid* controlling language, rewards, threats, external evaluation, and deadlines).	13 (93%)
• Using neutral language? (e.g. ‘may’ and ‘could’, and *avoid* ‘should’ or ‘must’).	14 (100%)
• Recognise barriers and conflicting feelings about wanting to be active.	12 (86%)
• Encouraging self-monitoring through use of pedometer (and other devices).	13 (93%)
• Encouraging setting time aside to include activity, and back up plans if this does not happen.	11 (78%)
Competence:	
• Discuss issues around exercising safely	11 (78%)
• Individualised goals for ability, and treatments.	9 (64%)
• Providing non-judgemental and positive feedback on progress.	14 (100%)
• Focusing on participants’ strengths and celebrate even the small goals.	13 (93%)
• Give support on how best to achieve goals.	9 (64%)
• Working through pros and cons of being physically active during/after treatment for breast cancer.	8 (57%)
• Help with ideas to overcome barriers for those during or after treatment.	6 (43%)
• Make sure that not achieving goals does not become a negative. Use it to explore any barriers and/or concerns to help improve the following week.	8 (57%)
Relatedness:	
• Value all opinions discussed. Do not judge progress by being negative or positive.	11 (78%)
• Acknowledging participants’ feelings and perspectives.	13 (93%)
• Giving positive feedback, such as their performance. Feedback must not make them feel they are being ‘tested’.	14 (100%)
• Help participants to indicate their reasons to change their activity levels.	7 (50%)
• Showing genuine appreciation and concern for participants by devoting time, energy and resources to support them to be physically active.	14 (100%)

Scores from seven participants were included in analysis of the Perceived Environmental Supportiveness Scale. The mean score was 90.71 (SD 13.4) (min 71 max 105).

### Physical activity dose

The PA dose delivered was assessed using FITT parameters calculated from participant diaries (Table 4). Eight out of 20 (40%) and nine out of 12 (75%) participants in Phases I and II, respectively, provided FITT data (i.e. completed a diary). Walking was the most common type of PA (57.8 and 72% in Phases I and II, respectively). Self-reported intensity was similar in Phases I and II. For all participants, mean intensity was 11.48, which is towards the high end of ‘light’ intensity of the Borg scale (range 6–20 with 20 being the hardest) [43]. Mean time in minutes for a PA session was 55.70 and 76.59 in Phases I and II, respectively. Only Phase II participants were given a pedometer to record step count; mean step count per day was 7584.56 (SD 3805.62).

**Table 4:** FITT parameters

	Phase I (*n* = 8)		Phase II (*n* = 9)	
	*N* (PA sessions)	%	*N* (PA sessions)	%
Total number of PA sessions reported by participants	313	–	710	–
Type % of *n*				
Walking		57.8%		72%
Jogging/running		6.4%		0.3%
Cycling		1.9%		0%
Other cardiovascular^a^		44.4%		16.8%
Resistance		17.3%		6.1%
Flexibility		2.2%		3.4%
Swimming		0%		0.8%
Housework		2.6%		17%
		Mean(SD)		Mean(SD)
Intensity reported by participants for each PA session (range 6–20 with 11 representing fairly light intensity requiring little or no effort)	286	11.97 (SD 2.54)	580	11.24 (SD 2.33)
Time (min) per day reported by participants for each PA session	310	55.70 (SD 54.07)	576	76.59 (SD 78.18)
			N (participants)	
Steps	–	–	9	75%
Average step count per day				7584.56 (SD 3805.62)

^a^E.g. spinning, running machine, aerobic classes

### Participant opinions about the intervention

Nine out of 20 in Phase I and 10 out of 12 participants in Phase II were interviewed. One interview was conducted by telephone, and the others were conducted face-to-face at the university.

#### Choice of PA programme

Four themes were identified from qualitative interviews about PA programme choice: travel distance, socialising, relevance and flexibility. In Phase II, the main reason for choosing telephone-delivered PA consultations rather than the leisure centre was long travel distance from the leisure centre. Nonetheless, travel distance created difficulties for participants choosing telephone-delivered PA consultations because they still needed to arrange a face-to-face PA induction with an exercise specialist at the leisure centre prior to commencing telephone-delivered support. One participant felt that travelling such a long distance just for a brief PA induction seemed hardly worth it. One participant who chose leisure centre with 3-month free membership found travelling frustrating and impeded regular attendance.

Some participants felt self-conscious about their appearance following mastectomy. These participants did not wish to engage in PA among the general public and therefore chose cardiac rehabilitation exercise classes (exercising with other people who were recovering from illness) or telephone-delivered PA consultations in Phase II. However, some participants chose the leisure centre with 3-month free membership in order to socialise. One participant, for instance, chose the leisure centre with 3-month free membership because she believed that it would help build her confidence to meet other people. Since her breast cancer diagnosis, she had spent most of the time meeting other people recovering from cancer and the leisure centre provided an opportunity to meet people who did not have cancer. One participant questioned the relevance of cardiac rehabilitation exercise classes for people with breast cancer since the programme was designed for people recovering from a cardiac event and chose the leisure centre with 3-month free membership. Some participants chose the leisure centre with 3-month free membership because it provided greater flexibility for being physically active. The leisure centre enabled people to engage in activity that was not weather dependent. Cardiac rehabilitation exercise classes were offered once a week, on a set day and time, which did not suit everyone. Concerns about missing sessions due to feeling unwell, for instance, were eased when it was explained that they could attend the leisure centre at a time that suited them.

#### Facilitators and barriers for engaging in PA

Four themes were identified from the qualitative interviews about facilitators and barriers for engaging in PA: feeling better, feeling ill, weight management, family and friends. Participants perceived that PA made them feel better. One participant said that she had felt powerless and helpless during her treatment for breast cancer and that engaging in PA gave her back a sense of control. Another participant said that when she was active it stopped her from worrying about ‘what might be’ (i.e. fear of recurrence). Nonetheless, a barrier to participation in the PA programme was feeling ill as a consequence of receiving adjuvant therapy. Some participants experienced fatigue or felt sick and dizzy which they said prevented them from partaking in PA. Several participants explained that they had put on weight due to treatments and wished to be active to help them to lose and maintain weight loss. Family and friends were key sources of support for engaging in PA. They accompanied participants during an activity such as walking. Having a pet dog also constituted a source of support because the dog needed to be walked. Family and friends acted as competition for daily step counts and were therefore an important source of motivation. Participants could become friends and help each other. A couple of participants who chose PA telephone-delivered consultations, for instance, teamed up to attend the gym together and found this was beneficial because they could talk about the exercises and how they were feeling on the day. Nevertheless, family commitments can also act as a barrier to participation. One participant explained that attending to the family members’ needs meant she was unable to go to the gym during a particular week.

## Discussion

The aim of this paper was to address the general lack of published data about how practitioners deliver a PA intervention and the extent to which participants engage in the PA programme [34]. This exploratory study raises the following issues relating to implementation of community-based PA programmes for people recovering from surgery for breast cancer:

### Reach and uptake

The study had very high retention rates with only two participants dropping out of the study due to ill-health. The retention rate is therefore somewhat consistent with previous trials of community-based PA interventions ≥ 80% [28, 45–48]. Nonetheless, the study consent rate (31%) is relatively low compared to other community-based PA interventions: 71% [45], 61% [49], 66% [46], 61% [47]. Why the study has a relatively low consent rate is unclear. The reasons given by eligible patients for refusing to participate in the study are similar to other studies and include health issues, not being interested, and other commitments (e.g. family and work) [45, 47, 49]. These factors are also cited in the literature as barriers to being physically active following a breast cancer diagnosis [27, 50–53]. Addressing these factors in future studies can therefore serve the dual purpose of increasing PA as well as the appeal of participating in studies of community-based PA programmes.

The eligibility rate (46%) is comparatively low compared to some previous trials of community-based PA interventions: 76.9% [45], 61% [49], 46% [46], 79% [47]. One seemingly obvious reason for this variation between studies is use of different eligibility criteria. Yet, two trials with similar eligibility criteria (e.g. people with breast cancer must have completed treatment, are physically inactive and have no contraindications for exercise) had differing eligibility rates of 42% [49] and 79% [45], respectively. Another possible explanation for variation in eligibility rates is a difference in clinician interpretation and application of eligibility criteria. In the study, the MDT excluded 45% of patients in Phase II but only 9% in Phase I, which could be an artefact of differences in recruitment time periods (3 versus 6 months) or clinician variation in application of eligibility criteria. The proportion of people excluded by clinicians will influence PA programme reach and convincing clinicians of the benefits of PA for people with cancer may go some way towards improving the recruitment rate [54].

### Engagement

The qualitative interviews give insight about patient PA programme choice, with some participants choosing telephone-delivered PA consultations because they did not wish to travel long distances to attend the leisure centre and others choosing telephone-delivered consultations because they felt self-conscious about their appearance following mastectomy and therefore did not wish to engage in PA in front of members of the public. Preference for home-based PA was found in a previous survey of rural people with breast cancer (*n* = 483), with respondents indicating a preference for home-based (63%), unsupervised (47%) and moderate intensity exercise (65%) that was primarily walking [55].

The PA intervention was designed to allow maximum participant choice for the type of PA that they did during the 12-week PA programme. Diaries completed by participants show that the most common type of PA was walking (57.8%) and intensity was ‘fairly light’ (mean = 11.97; SD 2.54). A survey of people with breast cancer (*n* = 160) during chemotherapy found that walking and exercises specific to women with breast cancer were most frequently performed and preferred [56]; another survey of patients (*n* = 23) [51] during treatment found that the majority preferred walking (100%) at moderate-intensity (61%) and another study (*n* = 12) found that walking was the most acceptable exercise modality [57]. Preferences for walking have also been found in a survey of cancer survivors with different diagnoses [58]. A challenge therefore is encouraging people to engage in their preferred activities, such as walking, at a level of intensity that will optimise health benefits and to progress mean rates of excursion during a 12-week programme. Addressing this challenge may be critical because the findings of observational studies of PA and breast-cancer specific and all-cause mortality that have been summarised in meta-analyses and systematic reviews suggest that there may be a dose-response [4, 59], and while there is limited evidence about a dose-response relationship for other health benefits, one meta-analysis revealed that there may be a dose-response relationship for fatigue [60].

### Fidelity

All Phase I participants received an induction by a cardiac rehabilitation physiotherapist or PA specialist from the leisure centre but only 50% of Phase II participants received an induction by a PA specialist from the leisure centre. In Phase II, use of each of the 22 SDT-techniques reported by the PA specialists during a telephone-delivered consultation was high, with only two techniques being used in half or less of the PA consultations delivered by telephone. In Phase II, participants perceived that their three basic psychological needs (autonomy, competence and relatedness) were met by the PA specialists. However, self-reported use of behaviour change techniques is limited because of biased reporting and objective measurement such as recording PA consultations should be considered in future trials. In the study, the average number of participant contacts (e.g. face-to-face, telephone, email) by a PA specialist over the 12-week PA programme was six. While this is half than what was planned, this actual number is similar or greater than other telephone-delivered PA trials of similar duration [45, 48]. In a previous trial, the mean number of calls was 6.7 (SD 1.81) [45], and in another study, the average total contact time over a 12-week home-based walking programme was 90 min [48]. Although the optimal contact time to promote behaviour change is uncertain, the qualitative interviews suggest that some participants found weekly ‘checking up’ by a PA specialist on their amount of PA a source of motivation. For these participants at least, weekly contact (face-to-face, telephone or email) over the course of a PA programme is likely to be important for the improvement of PA and health.

Nonetheless, a strength of the study is that it took place in a ‘real-world’ setting because it is delivered by PA specialists at a local leisure centre and therefore likely to reflect what would happen were the PA intervention implemented into the cancer care pathway. In the study, over half of the Phase II participants did not receive a face-to-face induction with a PA specialist whereas in a previous trial all face-to-face induction prior to telephone-delivered support were carried out [46]. A key difference between these two studies was who was delivering the intervention. In this study, the PA induction and telephone-delivered consultations were delivered by PA specialists at a community-based leisure centre who were expected to fit the delivery of the PA programme around their other daily tasks whereas in the other study the PA counsellor was a Master’s degree trained research assistant specifically employed to deliver the PA programme. This key difference may explain variation in intervention fidelity between the two studies. A challenge for using existing community-based services rather the research team delivering the PA intervention is therefore a lack of direct management over the weekly activities of the individuals who are delivering the intervention.

### Strengths and limitations

The strengths of this feasibility work relate to the recruitment of a sample generally representative of the breast cancer population and the evaluation of a PA intervention, which was delivered using a pragmatic approach suitable for translation into practice. Limitations include the study only being conducted in one site with a small sample which limits generalisability.

## Conclusion

The current community-based PA intervention is not yet suitable for progression to a definitive effectiveness randomised controlled trial. Further work is needed to optimise PA programme reach, uptake and fidelity. Further work is also required to assist participants to progress their level of exercise intensity when engaging in their preferred activity, for example, walking so that they achieve the health benefits associated with moderate to vigorous PA.

## Additional files

Additional file 1: **Figure S1.** Logic model for referral to PA programmes. (DOCX 91 kb)

Additional file 2: Quotations. (DOCX 111 kb)

Additional file 3: Flow chart. (DOCX 117 kb)

## Abbreviations

MDT: Multi-disciplinary team
PA: Physical activity
PAR-Q: Physical Activity Readiness Questionnaire
SDT: Self-determination theory
UK: United Kingdom
