# Supplementary material for: Physical activity referral to cardiac rehabilitation, leisure centre or telephone-delivered consultations in post-surgical people with breast cancer: a mixed methods process evaluation
Source: Pilot Feasibility Stud. 2018 Jun 1;4:108. doi: 10.1186/s40814-018-0297-1 (PMC5984397; doi:10.1186/s40814-018-0297-1)
Supplement: Supplementary file 1 — Figure S1. Logic model for referral to PA programmes. (DOCX 91 kb) [file 40814_2018_297_MOESM1_ESM.docx]

**Additional file 1: Figure S1 Logic model for referral to PA programmes**

| **Pre-PA programmes entry** | |  | **PA programmes (0-12 weeks)** | | | | | |  | **Longer-term outcomes** | | |
| --- | --- | --- | --- | --- | --- | --- | --- | --- | --- | --- | --- | --- |
| **Programme activity** | **Outcomes** |  | **Programme activity** | **Theory/BCTs** |  | **PA and –PA-related outcomes** |  | **Health and psychological outcomes** |  | **PA** |  | **Health** |
| Choice of PA programme offered to patients:  *Phase I:* cardiac rehabilitation or leisure centre;  *Phase II:* leisure centre or telephone-based PA consultations | Improve patient engagement with the PA programme by:  (1) Increasing ‘autonomy’ (feeling of being the origin of behaviour) for PA by providing choice of PA programme  (2) Remove barriers to PA (e.g. costs by short-term provision of free leisure centre membership; travel and distance by offering a telephoned-based PA programme) |  | Cardiac rehabilitation exercise class:   - 1:1 initial fitness assessment with incremental shuttle walk test by a cardiac physiotherapist and agreed exercises and intensities to complete during the classes - Class (15 min warm up; 20 mins cardiovascular and resistance/strength stations; 15 min cool down) once a week for 12 weeks - 1 hour health education sessions which took place after the exercise class and included general health advice (e.g. diet, exercise, relaxation), alongside cardiac specific sessions (e.g. medications). | Delivering the PA programme using BACPR guidelines such as:   - Goal-setting - Motivational interviewing techniques - Instruction e.g. showing how to perform a particular exercise or use equipment |  | (1) Increased self-efficacy for PA  (2) Increased PA |  | (1) Quality of life  (2) Fatigue  (3) Fear of recurrence |  | Maintenance of PA |  | (1) Reduced breast cancer recurrence risk  (2) All-cause mortality |
|  |  |  | Leisure centre:   - 1:1 initial induction with PA specialist to complete PA Readiness-Questionnaire (PAR-Q and agree on an individual exercise programme, including goal-setting - 3-month free leisure centre membership providing access to range of fitness classes, gym and swimming pool | Goal-setting  Health check |  |  |  |  |  |  |  |  |
|  |  |  | Telephone-based PA consultations:   - 1:1 initial induction with PA specialist to complete PAR-Q and agree an individual exercise programme, including goal-setting - 1:1 weekly telephone-delivered PA consultations with PA specialist based on Self-determination theoretical techniques (SDT) - Pedometer | - SDT-based PA programme using motivational interviewing techniques to foster autonomy, competence and relatedness - Pedometer to monitor step count |  |  |  |  |  |  |  |  |
