# Supplementary material for: Physical activity referral to cardiac rehabilitation, leisure centre or telephone-delivered consultations in post-surgical people with breast cancer: a mixed methods process evaluation
Source: Pilot Feasibility Stud. 2018 Jun 1;4:108. doi: 10.1186/s40814-018-0297-1 (PMC5984397; doi:10.1186/s40814-018-0297-1)
Supplement: Supplementary file 2 — Quotations. (DOCX 111 kb) [file 40814_2018_297_MOESM2_ESM.docx]

| **Theme** | **Quotation** |
| --- | --- |
| **PA programme choice** | |
| Travel distance | *“… just practicalities of getting there; if it had been at the bottom of the road I might well have been there more regularly” (P07:LC).* |
| Socialising | *'They’re nurses… It comes down to trust… because I knew I was going to lose my hair, I knew that people would probably know what was going on and I just wanted to be more in a, I don't know if it's like a safer environment …” (P02:CR)*  *“I'd lost confidence in from the time I got, stopped working, my confidence of going out and meeting people, doing things. I thought that would re-build by going to the leisure centre where I would maybe meet more people and meet people who wasn't actually recovering from cancer, but was there for enjoyment.” (P16: LC)*  *“And I'm still feeling uncomfortable and a bit unbalanced, to be honest at home I prefer to exercise without a bra or a prosthesis in, it's just more comfortable but I don’t feel quite right about doing that in the gym; now the leisure centre does have eh, well they have cubicles obviously in, in the main change rooms but you can get, eh request a private shower room if you want em, to be honest actually I, I haven't used it, it's been OK just using the cubicle, but I tend then to go home and shower rather than shower in, in the gym (P07:LC).*  *“After my mastectomy they give you the softy thing, which you're actually, your boob’s at your chin, the soft one, if you're a bit larger and, and I evidently seem to be, which I didn’t realise I was, and it was useless. So, going into the gym environment for some of us is very intimidating anyway but to go in when you've only got, quite obviously one, it's very noticeable when you've only got one [laughs], when you're wearing your jacket and stuff it's fine, but that's another reason for some of us wanting to do it exercises at home. So that you don’t have to be worried, you don’t have to be worried about, even though people probably aren’t looking at you, just the perception, because it's so obvious that people could be going “oh no, look at her,” and you get enough o' that, especially once you've lost your hair and stuff [slight laugh] without going into an environment where, even though it may be in your own head, but that's the perception you get. And that's why the option of doing it at home with the support was so appealing to me.” (P111)* |
| Relevance | *“I found out the cardiac route was really for people that had had serious heart problems, and the activity that they would be doing would be rather more sedate and more general eh...I thought the gym might be better for me” (P12:LC)* |
| Flexibility | *“...then I kind of thought well what if that one day is my day that I'm at chemo, then every three weeks I'm going to, to miss it, or what if it, you know, if you, if I feel really rough I can't sort of swap the days about, so I felt that the option at the Leisure Centre had more flexibility to it really.” (P10:LC)* |
| **Facilitators and barriers for engaging in PA** | |
| Feeling better | *“…… it is this business that one of the motivators for me to do the exercise is thinking well it does make me feel better afterward …immediately afterwards, there is a sort of immediate payback, and it was also the idea of symptom control, now I didn’t go and look at the literature or anything to see well, what's the basis for that, but I'm certainly thinking about the fluid retention and preventing lymphedema, em and just generally feeling better and keeping some sort of muscle tone …” (P07:LC)*  *“Em, I wouldn’t say it helped my fatigue because it got to be so bad for a while… but doing it made me feel better in my head, you know, I felt like I was doing something… overall, I think it has helped me overall but at the time of doing it I didn’t think oh I feel more energetic because of it, I didn’t, but I think my fatigue was quite severe.” (P103)*  *“It's getting rid o' that sense of, that sort of feeling of helplessness and powerlessness…. So this was a blessing because it gave you something to do, it gave you something, “right I can focus on this, this is somethin” (P109)*  *“I certainly think when I'm active I spend much less time thinking about what might be.” (P110)* |
| Feeling ill | *“You know it was the chemo that kind of started to wipe me out and then with the, changing onto the second type of chemo just, I, I just felt I, I couldn’t even get out my bed some days, you know I felt dizzy and sick getting up, I had blisters on my feet, my terrible sore haemorrhoid piles, you know em, and they all just em combined to make me think God, I can't walk ten yards never mind walk half an hour down there, do the exercises and, and come back kind of thing, and it pretty much, each month it's got, each chemo session it's got harder…” (P10:LC)*  *“Since this has happened, I mean I find I’ve very quickly tired and my breathing is absolutely appalling so I get to that point and I think ‘I’ve actually I’ve had enough.’ I’m not pushing myself as much as I know I should.” (P110)* |
| Weight management | *“I think yes, if I hadn’t gone then I would have, you know, possibly have put weight on anyway, but I possibly would have put more weight on that probably would have dented my confidence a bit. 'Cause there's so many physical changes that go on when you're going through treatment, you know...” (P01:CR)*  *“I'm on that, the Letrozole. I find I'm putting on weight and I think that keeps me focused to keep on top of things.” (P102)* |
| Family and friends | *“Having to attend some family matters eh I’ve had to put all these other things on hold… sometimes I haven’t been to the gym in the week eh, but I would like to continue to go” (P12:LC)*  *Interviewer: “Has it been helpful that you teamed up with someone that also had breast cancer?”*  *Participant: “Yes because we can em discuss it, em, you know what, what we're finding and I think, I think we can em*  *Interviewer: “What do you chat about?”*  *Participant: “Our exercises, how we're feeling, if we've been feeling alright all week.” (P102)*  *“Yeah, it [pedometer], you can get [slight laugh] very em competitive with yourself, and especially with my, my father-in-law does it, because he’s always quite active and he’s got an App on his phone, so he started just, you know: “oh, I've done this many steps,” I'd be like “well if he’s done that many steps, I've got to do …,” so you do …, and then my husband started as well, because on, you know he, his job can go from being very sedentary to very active, so some days he’d be like “Crikey, I've only done 4,000,” but, right, and you know, so then, so it's kinda made him, you know, maybe park the car a little further away, so it's benefitted everybody.” (P101)*  *“I've got two friends who walk with me and it tends to go, we go for an hour, sometimes a bit more, sometimes slightly less.” (P102)*  *“ I have to take him [pet dog] out, you know, and that was a, and I'm so glad we had him because I think if we didn’t it would have been a completely different story.” (P101)* |
