# Supplementary material for: Physical activity referral to cardiac rehabilitation, leisure centre or telephone-delivered consultations in post-surgical people with breast cancer: a mixed methods process evaluation
Source: Pilot Feasibility Stud. 2018 Jun 1;4:108. doi: 10.1186/s40814-018-0297-1 (PMC5984397; doi:10.1186/s40814-018-0297-1)
Supplement: Supplementary file 3 — Flow chart. (DOCX 117 kb) [file 40814_2018_297_MOESM3_ESM.docx]

Phase II screened prior to eligibility assessments

n=72

Drop out n=1(8%)

Drop out n=1 (5%0

Consenting n=20 (30%)

Cardiac rehab n=3 (15%)

Leisure Centre n=17 (85%)

Consenting n=21(32%)

Telephone n=9 (75%)

Leisure Centre n=3 (25%)

Phase I screened prior to eligibility assessments

n=68 (94%)

Phase I reasons for eligible patient non-participation n=25

Not interested n=8 (32%)

Back to normal PA n=2 (8%)

Travel distance to PA programme n=0 (0%)

Treatment side effects n=1 (4%)

Ill-health not related to breast cancer treatment n=4 (16%)

Other commitments e.g. back to work n=2 (8%)

Missing data n=8 (32%)

Phase I reasons for eligible patient non-participation n=47

Not interested n=8 (17%)

Back to normal PA n=6 (13%)

Travel distance to PA programme n=8 (17%)

Treatment side effects n=8 (17%)

Ill-health not related to breast cancer treatment n=4 (9%)

Other commitments e.g. back to work n=3 (6%)

Missing data n=10 (21%)

Phase I eligibility n= 37 (54%)

Reasons for ineligibility:

MDT decided unsuitable for inclusion n = 14 (45%)

*Reasons:*

*Clinically not stable n=1*

*Inpatient n=1*

*Heart failure n=1*

*COPD n=1*

*Complex medical history n = 1*

*Other carcinoma n = 2*

*Cerebrovascular disease n=1*

*Poor wound healing n=1*

*Other medical condition with treatment required n=2*

*Missing data n=3*

Cognitive impairment n = 1 (3%)

Further surgery n = 7 (23%)

Benign tumour n = 9 (29%)

Travel distance n = NA

No access to a landline or mobile phone n = 0 (0%)

Phase I eligibility n= 67 (42%)

Reasons for ineligibility:

MDT decided unsuitable for inclusion n = 8 (9%)

Cognitive impairment n = 0 (0%)

Further surgery n = 10 (11%)

Benign tumour n = 16 (17%)

Travel distance n = 57 (63%)

No access to a landline or mobile phone NA

Phase I screened prior to eligibility assessments

n=158 (100%)

Phase I screened prior to eligibility assessments

n=158
